# The association among SES, screen time, and outdoor play in children at different ages: The GECKO Drenthe study

**DOI:** 10.3389/fpubh.2022.1042822

**Published:** 2023-01-10

**Authors:** Congchao Lu, Rikstje Wiersma, Eva Corpeleijn

**Affiliations:** ^1^School of Public Health, Tianjin Medical University, Tianjin, China; ^2^Tianjin Key Laboratory of Environment, Nutrition and Public Health, Tianjin, China; ^3^Center for International Collaborative Research on Environment, Nutrition and Public Health, Tianjin, China; ^4^Department of Epidemiology, University Medical Center Groningen, University of Groningen, Groningen, Netherlands

**Keywords:** socioeconomic inequalities in children, Equivalized Household Income Indicator, screen time (ST), outdoor play, birth cohort study

## Abstract

**Introduction:**

This study examined the association among socioeconomic status (SES), screen time, and outdoor play in children at different ages in the GECKO Drenthe birth cohort study.

**Methods:**

Valid data were obtained from two surveys at ages 3–4 years and 10–11 years. Screen time (TV watching and computer use) and outdoor play were reported by parents. Childhood SES was derived by a synthetic “Equivalized Household Income Indicator,” an estimated disposable income. Quantile regression models (cross-sectional analysis) and linear regression models (change between 3–4 and 10–11 years) were used.

**Results:**

In general, screen time increased strongly from a median of 51 min/day at 3–4 years (*n* = 888) to 122 min/day at 10–11 years (*n* = 1023), whereas time spent on outdoor play remained stable over age (77 min/day at 3–4 years and 81 min/day at 10–11 years). More time spent on outdoor play (50th quantile) was found in children with low SES families at 3–4 years, while at 10–11 years, more outdoor play was found in the high SES group. At 10–11 years, in the higher ranges of screen time, children from high SES had relatively lower screen time [50th quantile: −10.7 (−20.8; −0.6); 75th quantile: −13.6 (−24.4; −2.8)]. In the longitudinal analysis (*n* = 536), high SES was associated with an increasing time spent on outdoor play [11.7 (2.7; 20.8)].

**Conclusion:**

Socioeconomic disparities in children's outdoor play and screen behavior may be more obvious with increasing age. Low SES may facilitate both outdoor play (at 3–4 years) and screen time (at 10–11 years); however, children from high SES families develop slightly more favorable behavior patterns with age.

## 1. Introduction

Human behaviors and activities are changed due to the revolution of information technology. For example, digital communications have influenced how people work, study, and spend their leisure time. In most countries, children are spending greater time engaged in screen-based entertainment, such as television and computers. It is well-recognized that excessive screen time is associated with an increasing trend of physical inactivity in most societies around the world (1). Furthermore, increasing screen time is associated with poorer sleep outcomes (2) and delayed motor development skills in preschool children (3), and with increased obesity risk in children and adolescents (4, 5).

The health benefits of outdoor play have been emphasized by several researchers in terms of reducing myopia and developing motor skills, along with improving social skills (6). In addition, encouraging outdoor play might prove to be an effective strategy in children for curbing physical inactivity, since it is a cheap and natural way (7). A study indicated that every additional hour spent outdoors per day was associated with 7 min less sedentary time on an average day among Dutch preschoolers (8). Meanwhile, evidence indicated that outdoor play has been replaced by more time using electronic media indoors (9, 10). The World Health Organization guidelines state that preschool children (3–4 years old) should spend no more than 1 h of screen time each day (11), whereas children and adolescents (5–17 years old) should limit the amount of recreational screen time (12). Due to the co-dependence of lifestyle behaviors, an increase in one behavior would be expected to result in a decline in another (13). However, few studies have reported the co-dependence of the relationships between changes in screen time and changes in outdoor play. Understanding the long-term changes in those behaviors throughout childhood contributes to the early evidence-based planning of public health interventions. Thus, it needs to be understood how screen time and outdoor play change with age.

As lifestyle behaviors, outdoor play, and screen behaviors depend on their societal context, socioeconomic status (SES) which represents the social, cultural, and economic features of a family is an important factor that affects opportunities for these behaviors (14). Assessing SES in early life is also essential to control for confounders when studying outcomes that are strongly socially shaped (15). Studies investigated the association between SES and screen time, and inconsistent results may be derived because various SES indicators were used (16). For example, there can be marked racial differences in income at a given educational level (17). Thus, caution is needed when evidence based on different single SES indicators were synthesized for informing social policy design to effectively reduce health disparities in a socially diverse society (18). However, accurately measuring family income through questionnaires is difficult due to several issues, and it is essential to have harmonized comparable SES indicators over different studies. In this study, a standardized income indicator, the “Equivalized Household Income Indicator (EHII)” that measures the equivalized disposable household income based on a cluster of indicators (including but not limited to maternal and paternal education level, housing type, and family size) was used as children's SES indicator (19). We are aiming to explore SES differences in screen time and outdoor play at different ages, and the changes in screen time and outdoor play between ages 3–4 and 10–11 years in the Groningen Expert Center for Kids with Obesity (GECKO) Drenthe birth cohort.

## 2. Methods

### 2.1. GECKO Drenthe birth cohort

Data were derived from the GECKO Drenthe birth cohort, which focuses on the development of overweight and obesity in children living in Drenthe, a northern province of the Netherlands. Details of the study have been reported elsewhere (20). All mothers of children born between April 2006 and April 2007 and living in Drenthe were invited to participate during the third trimester of their pregnancy. Almost 3,000 pregnant women were recruited. Monitoring of the children started in the last trimester of the pregnancy and is still ongoing. At the age of 10–11 years, 2,299 children were measured for follow-up.

### 2.2. Data collection

**Figure 1:**
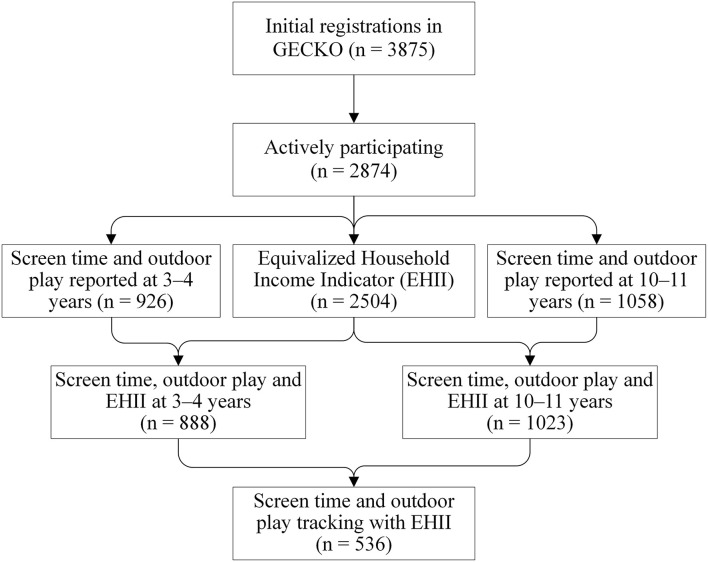
Flowchart of the participants in the GECKO Drenthe study.

At baseline, child and family information, including birth dates of family members, parental country of birth, parental educational levels, parental occupational status, mother living with a partner, and dwelling type, was collected. When children were aged 3–4 years (2010–2011) and 10–11 years (2017–2018), the height and weight of children were measured by trained preventive child healthcare nurses as part of a regular health screening. Children's overweight and obesity aspects were classified according to the age-specific and gender-specific cutoffs of Cole and Lobstein (21). Questionnaires for parents when the child was 45 months were handed out during visits to the Well Baby Clinic, and questionnaires for children of 11 years were sent to the parents by post. Children who had valid data on screen time and outdoor play both at 3–4 years and/or 10–11 years and the synthesized indicator of household income estimation were selected as potential participants for this study. The flowchart of the participants used in this study is shown in Figure 1.

### 2.3. Socioeconomic status

Childhood SES in this study was defined by the “Equivalized Household Income Indicator (EHII),” which is a standardized, cross-cohort comparable income indicator developed by Pizzi et al (19). The EHII was derived by a prediction model combining household and personal variables to estimate the disposable household income. The following household and personal variables were included in the prediction model: “maternal age,” “maternal educational level,” “maternal occupational status,” “maternal country of birth,” “paternal age,” “paternal educational level,” “paternal occupational status,” “paternal country of birth,” “cohabitation status (living with/without a partner),” “dwelling type,” and “family size.” The prediction model was constructed using external data of the Netherlands from 2011 from the pan-European Union Statistics on Income and Living Conditions (EUSILC) surveys (22) and validated with data from 2015. The prediction model had a good overall performance (R^2^ = 0.455), and details have been reported elsewhere (23). The derived household income was interpreted as the log equivalized monthly total disposable household income that a person with those characteristics would have had in 2011. The values of the EHII back-transformed monthly total disposable household income are equal to 729 Euro−2943 Euro in this study. A positive association was found between the estimated disposable income by EHII and self-reported household income in 2,212 participants in the cohort (*r*_s_ = 0.428, *p* < 0.001). Childhood SES was categorized into low SES (<1,717 Euro/month), middle SES (1,717–2,172 Euro/month), and high SES (>2,172 Euro/month) based on tertiles of the estimated household income.

### 2.4. Screen time and outdoor play

Parents/guardians reported the frequency and duration of their children's television (TV) time, computer time, and outdoor play, considering a typical week in the past month, both when the child was 3–4 years old and 10–11 years old. In those questionnaires, TV time was defined as time spent watching TV, video, or DVD, and computer time was defined as time spent using a game computer. Child outdoor play was defined as time spent playing outside, with questions based on a study by Aarts et al. (24). For example, parents were asked to report the duration of outdoor play during weekdays (response categories ranged from 0 to 5 days) and weekend days (response categories ranged from 0 to 2 days), with the answer options (no outdoor play, <30 min per day, 30 min−1 h per day, 1–2 h per day, and more than 2 h per day). To sum up the result, “no outdoor play” was recorded as 0, “ <30 min” was recorded as 15 min, “30 min−1 h” was recorded as 45 min, “1–2 h” was recorded as 90 min, and “more than 2 h” was recorded as 150 min. The average number of minutes per day was computed to obtain an overall outdoor play time average per day. TV time and computer time were asked and recorded in the same way, separately, and they were summed together as screen time. The outcomes were screen time (min/day) and outdoor play (min/day), both at 3–4 years and 10–11 years, and the changes in screen time (min/day) and outdoor play (min/day) between the two surveys are referred to as tracking data.

### 2.5. Parents' rules on child screen use and outdoor play

Parents' rules on “the duration” of child TV watching and game computer use were asked separately in questionnaires, and those rules were combined to make rules about “the duration” of screen use. For example, if there was a rule at home “about the duration of the child be allowed to watch TV” and/or there was a rule “about the duration of the child be allowed to use the game computer,” then it was defined as there was a rule at home “about the duration of child's screen use.” In the questionnaire, there were also rules about “the duration” of the child's outdoor play. All of those rules were asked at both 3–4 years and 10–11 years.

### 2.6. Statistical analysis

Continuous variables were presented as means with SDs or, if data were skewed, as the median with 25th−75th percentile. Categorical variables were presented as rates in number and percentages. To examine the differences in characteristics between children with tracking data included in the analyses to children lacking tracking data, a *t*-test was used for normally distributed continuous variables, and the Mann–Whitney *U*-test was used for non-normal distributed continuous variables. Differences in categorical variables were tested by the chi-square test. The cross-sectional correlation between screen time and outdoor play was checked by Pearson correlation (normal distribution), or Spearman's correlation was used if data were skewed. To determine the cross-sectional relationships among SES, child screen time, and outdoor play in both surveys, quantile regression analysis was used, with children from middle SES families set as the reference group. Regression coefficient estimates at the 25th, 50th, and 75th quantiles of each outcome were reported, and all models were adjusted for age, sex, and parents' rules on child screen use/outdoor play. The season was not added in those models, since there was no difference in the season of measurement between the three SES groups (survey of 3–4 years: χ^2^ = 7.388, *p* = 0.286; survey of 10–11 years: χ^2^ = 9.747, *p* = 0.136). To determine the relationships between SES and the changes in screen time and outdoor play between the two surveys, the linear regression analysis adjusted for age and sex was used. For the sensitivity analyses, the maternal level of education in three categories was checked as an indicator of SES by the same methods, with children whose mothers were at the middle educational level as the reference group. IBM SPSS Statistics V.26 for Windows was used for this study, with test level α = 0.05, and analyses were conducted in 2022.

## 3. Results

In the survey of 3–4 years, 926 children had valid questionnaire data on screen time and outdoor play, and this number is 1,058 at 10–11 years. Combined with SES information, 888 children (52.9% boys) at 3–4 years and 1,023 children (48.9% boys) at 10–11 years had valid data for cross-sectional analysis. Of these, 536 children (53.7% boys) had valid data on screen time and outdoor play tracking (Figure 1). Missing data from the questionnaire at 3–4 years were mainly attributable to logistical and organizational problems.

Children with data on screen time and outdoor play tracking showed a higher level of maternal education (*n* = 208, 38.8% of total 536) compared to children without tracking data (*n* = 116, 33.0% of total 352; χ^2^= 91.274, *p* = 0.000). As shown in Table 1, screen time at 3–4 years consisted mostly of TV time, and it increased from the median of 51 (39; 90) min/day to 122 (90; 165) min/day at 10–11 years. The time children spent playing outside did not change too much as children got older. More outdoor play was associated with less screen time in both surveys (ρ = −0.098, *p* < 0.001; *r* = −0.098, *p* < 0.001).

**Table 1:** Characteristics of the study population in the GECKO Drenthe cohort study.

**Characteristics**	**3–4 years (*n* = 888)**	**10–11 years** **(*n* = 1,023)**
Sex		
Female	418 (47.1%)	523 (51.1%)
Male	470 (52.9%)	500 (48.9%)
Age (years), mean (SD)	3.9 (0.2)	11.1 (0.4)
Body weight status		
Normal weight/underweight	693 (78.0)	855 (83.6)
Obesity and overweight	64 (7.2)	146 (14.3)
Missing	131 (14.8)^*^	22 (2.2)
Screen time (min/day), median (25th; 75th)	51 (39; 90)	122 (90; 165)
TV time (min/day)	45 (39; 90)	75 (51; 107)
Game computer time (min/day)	0 (0; 2)	45 (24; 90)
Outdoor play (min/day), median (25th; 75th)	77 (45; 107)	81 (49; 107)
Rules about the duration screen using		
Yes	620 (69.8)	641 (62.7)
No	252 (28.4)	243 (23.8)
Missing	16 (1.8)	139 (13.6)
Rules about the duration of outdoor play		
Yes	262 (29.5)	305 (29.8)
No	619 (69.7)	579 (56.6)
Missing	7 (0.8)	139 (13.6)

* Random missings due to logistical reasons.

As shown in Table 2, the cross-sectional associations of SES, children's screen time, and outdoor play were analyzed by quantile regression analysis, with children from middle SES families set as the reference group. Regression coefficient estimates at the 25th, 50th, and 75th quantiles cut-points of each outcome are given in both surveys. Compared to the middle SES group, more time spent on outdoor play was found in children with low SES families at the 50th quantile in the survey of 3–4 years. At the same time, more time spent on outdoor play was found in children with high SES families at the 50th quantile in the survey of 10–11 years. A pattern of less time spent on screen time at higher quantiles was observed in the high SES group in the survey of 10–11 years; the regression coefficient estimate at the 50th quantiles was −10.7 (95% CI: −20.8; −0.6), and at the 75th quantile, it was −13.6 (95% CI: −24.4; −2.8). This indicates that when children watch a little TV, the SES differences are negligible, whereas the difference becomes more obvious in the higher ranges of screen time.

**Table 2:** The estimated regression coefficients in screen time and outdoor play according to the level of socioeconomic status.

**Potential determinants**		**3–4 years (min/day**, ***n*** = **888)** β **(95% CI)**		**10–11 years (min/day**, ***n*** = **1023)** β **(95% CI)**	
		**Screen time**	**Outdoor play**	**Screen time**	**Outdoor play**
Quantile 0.25	Low SES	2.9 (−4.8; 10.5)	−2.1 (−9.9; 5.7)	−4.5 (−16.6; 7.7)	−1.1 (−10.8; 8.5)
	High SES	1.4 (−5.5; 8.4)	5.4 (−1.7; 12.4)	−3.0 (−12.7; 6.6)	4.4 (−3.3; 12.2)
Quantile 0.50	Low SES	−3.6 (−11.0; 3.8)	**12.9 (3.5; 22.2)**	−4.3 (−17.0; 8.4)	4.9 (−4.6; 14.5)
	High SES	−4.3 (−11.0; 2.4)	0.0 (−8.5; 8.5)	**−10.7 (−20.8; −0.6)**	**8.5 (0.9; 16.2)**
Quantile 0.75	Low SES	−3.2 (−13.2; 6.8)	12.9 (−0.5; 26.2)	1.4 (−12.2; 15.0)	0.0 (−14.0; 14.0)
	High SES	−3.2 (−12.3; 5.9)	−0.0 (−12.1; 12.1)	**−13.6 (−24.4; −2.8)**	0.0 (−11.1; 11.1)

Bold: *p* < 0.05. Quantile regression analyses were used, and all models were adjusted for age, sex, and parental rules about the duration of the child's screen-using/outdoor play. The models show the differences in screen time and outdoor play for SES groups (middle SES as reference) for a given level of screen time or outdoor play, based on quartiles. For example, at the 75% percentile level of screen time, at 10–11 years of age, children from families with high SES had 15 min of screen time less per day than children from families with middle SES.

**Figure 2:**
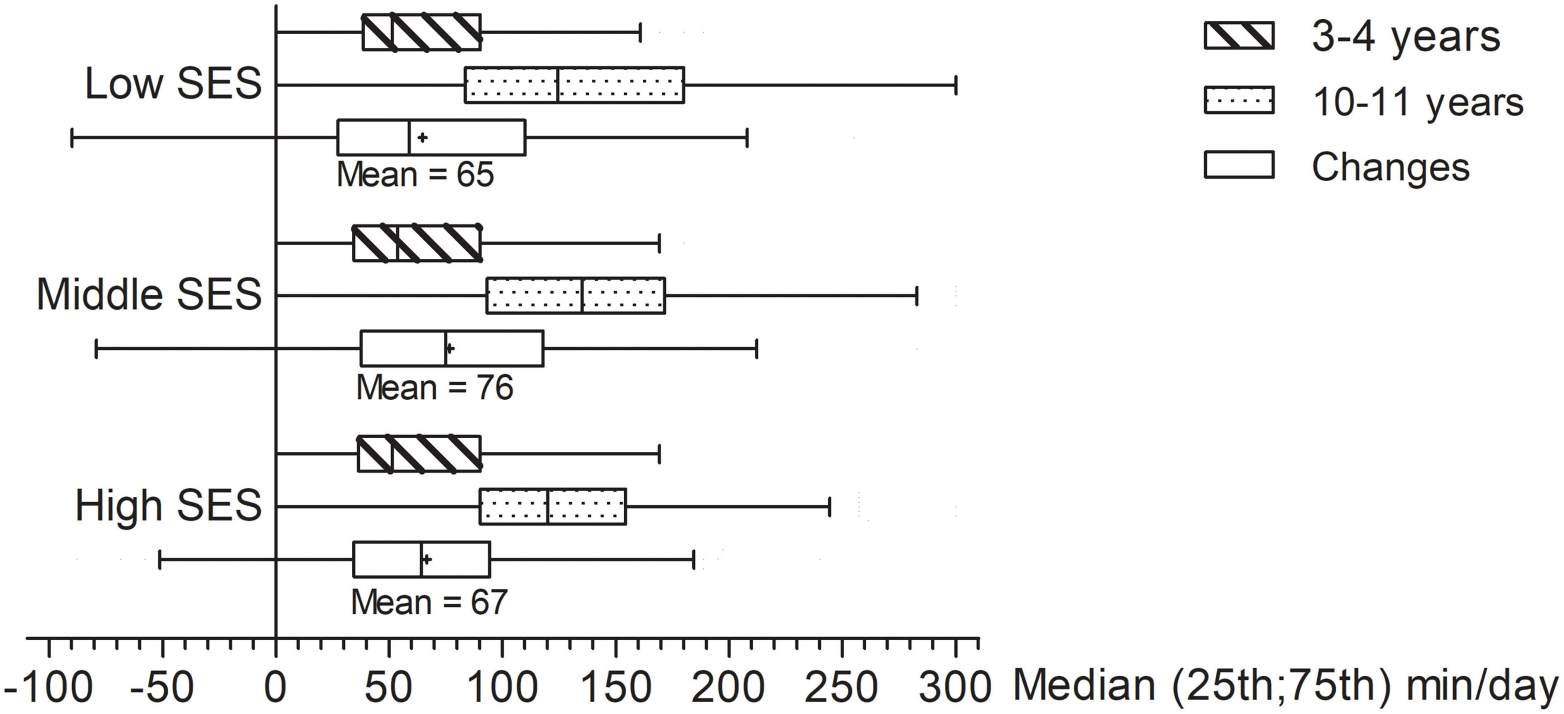
Daily screen time at different ages, and changes in screen time, according to socioeconomic status.

**Figure 3:**
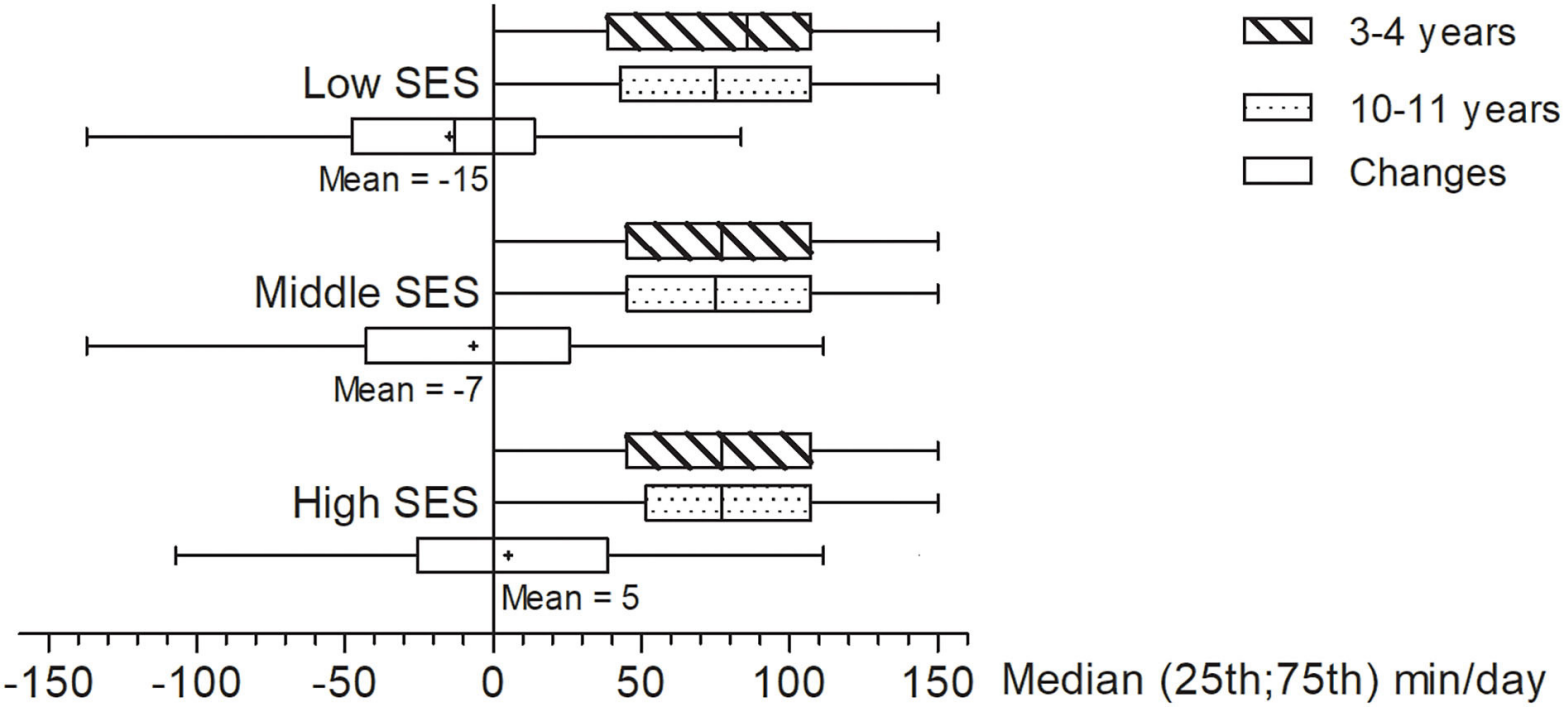
Daily time spent on outdoor play at different ages, and changes in outdoor play, according to socioeconomic status.

The associations of SES and changes in children's screen time and outdoor play are given in Table 3. The average of changes in children's screen time in low, middle, and high SES groups were 65 (95 CI of mean: 52; 77), 76 (68; 85), and 67 (60; 73) min/day, respectively (Figure 2). It also showed that boys showed a greater increase in time spent on screen behaviors compared to girls. There was no difference in changes in screen time between SES groups, adjusted by sex and age. For changes in time spent on outdoor play, older children were observed to spend slightly less time on outdoor play at 10–11 years compared to when they were younger (Figure 3). The mean change was −15 (−25; −5) min/day in the low SES group, −7 (−14; 0) min/day in the middle SES group, and 5 (−1; 11) min/day in the high SES group (Figure 3). It showed that high SES was associated with maintenance and even a slight increase in time spent on outdoor play [β and 95% CI: 11.7 (2.7; 20.8)], compared to middle SES and low SES groups. As children from low SES families used to have slightly more time spent in outdoor play (Table 2), and outdoor play was maintained and even slightly increased in children from high SES families (Table 3), the eventual time spent in outdoor play at 10–11 years of age was comparable between groups (Table 2).

**Table 3:** Socioeconomic status and changes in children's screen time and outdoor play from 3–4 to 10–11 years.

**Potential determinants**	**Descriptive data** ***n*** **(%)/mean (SD)**	**Changes in screen time (min/day)** **β (95% CI)**	**Changes in outdoor play (min/day)** **β (95% CI)**
Sex	0 = female, *n* = 248 (46.3%); 1 = male, *n* = 288 (53.7%)	**16.8 (7.3; 26.2)**	−1.7 (−9.9; 6.5)
Age at 10–11 years	11.1 (0.4) (years)	9.7 (−1.7; 21.1)	**−16.7 (−26.5; −6.9)**
Socioeconomic status (SES)	Middle SES as reference, *n* = 189 (35.3%)		
	Low SES, *n* = 94 (17.5%)	−10.5 (−24.3; 3.2)	−7.6 (−19.5; 4.3)
	High SES, *n* = 253 (47.2%)	−8.3 (−18.8; 2.2)	**11.7 (2.7; 20.8)**

Bold: *p* < 0.05.

To determine the co-dependence of the relationships between changes in screen time and changes in outdoor play between the two surveys, linear regression analysis adjusted for age, sex, and SES was used. It showed that a decrease in screen time {β and 95% CI: −0.1 [−0.2; (−0.1)]} was associated with increasing outdoor playtime between the two surveys.

The results of the sensitivity analyses using maternal education level as an alternative indicator of socioeconomic position are given in Supplementary Table 1. It showed that the findings were materially unchanged. However, no different findings were found for time spent on outdoor play at 10–11 years, or changes in screen time or outdoor play between children in different maternal educational level families (Supplementary Table 2).

## 4. Discussion

This study examined how screen time and outdoor play depend on SES at different ages in the GECKO Drenthe birth cohort study. In general, children's screen time increased strongly with age, whereas time spent on outdoor play did not change too much as children got older. At 3–4 years, more time spent on outdoor play was found in children with low SES families. At 10–11 years, more outdoor play was found in children with high SES families, and a pattern of less time spent on screen time at higher quantiles was also observed in the high SES group. In addition, as an indicator of SES, the EHII captured longitudinal variations in socioeconomic inequalities in child outdoor play.

In this study, 38.9% of children spent more than 1 h of screen time per day at 3–4 years of age, while 54.2% of children spent more than 2 h per day of screen time at 10–11 years of age. Interestingly, an international study of 10-year-old children reported a similar percentage (54.2% of 5,844 children) of children who spent more than 2 h of screen time per day from 12 countries around the world (25). Over the past century, people in most countries have gotten used to new lifestyles based on lack of PA, increased nutritional consumption, and have shifted away from nature's 24-h day/night rhythm through the development of artificial light, such as screens. In the meantime, there is a growing concern about the adverse impact of screen behaviors on children's health, including adiposity, unhealthy diet, and depressive symptoms (26). For example, the latest systematic review has summarized that there was strong evidence for the association between home media environment and adiposity in childhood (≤12 years) (27). Although the etiology of media exposure and adiposity is still unclear, evidence indicated that exposure to the light of shorter wavelengths by screen usage might impact human metabolism (28). Furthermore, as home media use continues to evolve, it is necessary to explore if children are at high risk of adverse screen behaviors based on their socioeconomic group. Because screen habits may track from as early as infancy (29), early childhood could be an opportune time to intervene to reduce excessive screen time (30). Previous studies found that higher SES was inversely associated with high screen time in children (29, 31). As we found in this study, the difference in SES becomes more obvious in the higher ranges of screen time. At the same time, many children from high SES families were also at risk of high screen time with increasing age. This indicated that screens have become more widely available to all families with various economic conditions. As differences in screen time were much larger within SES groups than between SES groups, our result emphasized the potential need for interventions on reducing screen time in most children irrespective of socioeconomic group.

Active outdoor play for children's health and development is of particular importance since it could improve children's wellbeing in the physical, emotional, social, and cognitive domains (32). This study showed that more time spent on outdoor play was found in children with low SES families in the survey of 3–4 years, compared to the middle SES group. For preschool children, outdoor time could be a good opportunity for children to be active (33). Outdoor play may be more attractive to those parents from low SES families, since it is cheap and natural, and young children are highly dependent on their parents to create opportunities for activities. Another possible explanation is that parents from low SES families may have more free time to accompany their children to play outside (34). For example, a higher proportion of unemployed mothers (25.1%) was found in the low SES group in this study, compared to the middle (6.3%) or high (*n* = 0) SES groups. We re-analyzed the data to test this hypothesis, and found that more time spent on outdoor play was found in children with low SES families at the 50th quantile [18.0 (6.4; 29.6)] and 75th quantile [30.0 (13.4; 46.6)] on weekdays, but not on weekends. However, the older child may have determined the frequency at which they played outdoors themselves because they have developed the ability of independent mobility, for example, going outside themselves and spending time playing outdoors around their communities (35). A systematic review found that children play outdoors more when there is less traffic, increased neighborhood greenness, and when they have access to a yard (ages 2–15 years) (36). One study found that Canadian children (10.2 ± 1.0 years) living in lower SES areas had a lower level of outdoor play on weekend days compared to their peers from higher SES areas (37). Perhaps high SES neighborhoods may facilitate children to play outside, for example, in their private yards. This could be the reason that children from families with higher SES spent more time on outdoor play in the survey of 10–11 years. Furthermore, we cannot exclude that the social desirability bias affected the responses in some way (38); since the responses of parents from low SES families during the survey of 10–11 years (19.6%) were lower than in the survey of 3–4 years (26.5%).

It is speculated that the time young people spend outdoors had declined dramatically with age because of engaging in screen-based behaviors indoors (25, 39). In this study, data showed that every additional 10 min more of screen time per day was associated with 1 min less of outdoor play. This indicated that the increase in screen time can only be explained by changes in outdoor play to a very limited extent. Although, in our study, we did not observe a dramatic decline in outdoor play with age in other studies. There may be some other reasons for the decreased outdoor play, such as increased academic pressures in school children (40). Furthermore, some researchers concluded that more screen time might be related to a lack of appealing nearby play opportunities or community destinations. One study of the GECKO Drenthe birth cohort found that families living in a supportive neighborhood might have more opportunities to be physically active, and they were more likely to make use of these facilities for themselves and their children (8). In addition, neighborhood outdoor play environments may vary by socioeconomic position, for example, a systematic review summarized that there was a positive association between area-level socioeconomic position and green space (41). Thus, future studies need to identify barriers and facilitators which appeal to children's interests that can help to enhance outdoor time and lower screen time in different socioeconomic areas.

In this study, we used the EHII as a synthetic SES indicator and captured longitudinal variations in socioeconomic inequalities in child outdoor play. This indicated that the EHII seems more sensitive to longitudinal changes in child behaviors, compared to other SES indicators, for example, maternal education (42). Furthermore, the EHII is of potential interest because it is expected to vary over time, capturing changes in SES (19). Moreover, household income can change over time but is difficult to estimate through questionnaires because of non-response. In aiming at reducing the intergenerational transmission of inequalities in socioeconomic resources, the identification of inequalities in health behaviors between different socioeconomic backgrounds in childhood is essential (43). Therefore, a standardized and comparable SES indicator is important for harmonizing comparable socioeconomic positions across populations and time. This is particularly relevant in the context of international collaborative studies (15), for example, combining data in birth cohort studies aiming at identifying risk factors leading to disease across the lifecycle across countries (44). Currently, the availability of the EHII as a marker of SES is increasing in European birth cohort studies, and more studies are needed to examine the availability of the EHII in future (19).

An important strength of this study was that the evidence was based on a birth cohort study, with a good representativeness population for the socioeconomic position (20). This child SES indicator uses multiple domains of SES, and the SES groups can be compared with other collaborative European birth cohort studies. Quantile regression analysis was used, which is an effective statistical method when the health outcomes by proxy report do not follow a normal distribution (45). This means that associations with SES could be studied for different absolute levels of screen time and outdoor play. Parents' rules on screen time and outdoor play were used to adjust for the analysis, since parental regulation may be an important behavioral determinant of a child's outdoor play (46) or screen use (47). A limitation is that due to incomplete data during follow-up, a considerable number of cases could not be included in this analysis. However, the selection bias was small, with a slight bias toward higher-educated families but still more representative of lower SES than in other birth cohorts. In addition, child screen time and outdoor play were reported by parents, and those outcomes may be overestimated or underestimated especially when children were more independent at an older age.

## 5. Conclusion

In conclusion, this study indicated that the socioeconomic disparities in children's outdoor play and screen behavior may be more obvious with age. Low SES may facilitate both outdoor play (at 3–4 years) and screen time (at 10–11 years). However, children from high SES families develop slightly more favorable behavior patterns with age. Therefore, special attention should be paid to early health interventions for children from low SES families, especially aiming at reducing screen time. Furthermore, the ‘Equivalized Household Income Indicator' as a child SES indicator seems more sensitive to reflect the socioeconomic disparities in changes in children's behaviors with increasing age, compared to maternal education.

## Data availability statement

The raw data supporting the conclusions of this article will be made available by the authors, without undue reservation.

## Ethics statement

The studies involving human participants were reviewed and the study was approved by the Medical Ethics Committee of the University Medical Center Groningen (UMCG), in accordance with the 1975 Declaration of Helsinki (as revised in 1983). Written informed consent to participate in this study was provided by the participants' legal guardian/next of kin.

## Author contributions

EC, CL, and RW designed the study and were involved in the interpretation of data. CL performed the analysis and drafted the manuscript. All authors were involved in the revision of the manuscript and approved the final version of the submitted manuscript.
