# Supplementary material for: The association among SES, screen time, and outdoor play in children at different ages: The GECKO Drenthe study
Source: Front Public Health. 2023 Jan 10;10:1042822. doi: 10.3389/fpubh.2022.1042822 (PMC9872556; doi:10.3389/fpubh.2022.1042822)
Supplement: Supplementary file 1 [file Table_1.docx]

Additional table 1. The estimated regression coefficients in screen time and outdoor play according to the level of maternal education.

| Potential determinants | | 3–4 years (min/day, n = 888)  β (95% CI) | | 10–11years (min/day, n = 1023)  β (95% CI) | |
| --- | --- | --- | --- | --- | --- |
|  |  | Screen time | Outdoor play | Screen time | Outdoor play |
| Quantile 0.25 | Low level | −7.2 (−14.6; 0.1) | −1.3 (−9.2; 6.6) | −7.8 (−20.4; 4.9) | 2.9 (−6.5; 12.3) |
|  | High level | −6.4 (−13.2; 0.4) | −2.1 (−9.4; 5.1) | −7.3 (−17.4; 2.7) | −0.2 (−7.8; 7.3) |
| Quantile 0.50 | Low level | −4.3 (−11.3; 2.7) | **12.9 (3.6; 22.1)** | −6.4 (−18.9; 6.1) | 5.5 (−4.0; 15.0) |
|  | High level | −4.3 (−10.8; 2.2) | −0.0 (−8.4; 8.4) | **−15.0 (−24.9; −5.1)** | −0.3 (−8.0; 7.3) |
| Quantile 0.75 | Low level | −5.9 (−15.8; 3.9) | **17.1** (**3.9; 30.4)** | 0.0 (−12.8; 12.8) | 0.0 (−13.7; 13.7) |
|  | High level | −8.1 (−17.2; 1.0) | −8.6 (−20.6; 3.5) | **−18.7 (−28.8; −8.5)** | −0.0 (−11.1; 11.1) |

*Bold: p value<0.05. All models were adjusted for age, sex, and parental rules about the duration of child’s screen using / outdoor play.*
