# Supplementary material for: The association among SES, screen time, and outdoor play in children at different ages: The GECKO Drenthe study
Source: Front Public Health. 2023 Jan 10;10:1042822. doi: 10.3389/fpubh.2022.1042822 (PMC9872556; doi:10.3389/fpubh.2022.1042822)
Supplement: Supplementary file 2 [file Table_2.docx]

Additional table 2. Maternal education and changes in children’s screen time and outdoor play from 3-4 years to 10-11 years.

| Potential determinants | Descriptive data  n (%) / mean (SD) | Changes in screen time (min/day)  β (95% CI) | Changes in outdoor play (min/day)  β (95% CI) |
| --- | --- | --- | --- |
| Sex | 0 = female, n = 248 (46.3%);  1 =male, n = 288 (53.7%) | **18.0 (8.5; 27.4)** | 1.9 (−6.3; 10.1) |
| Age at 10–11 years | 11.1 (0.4) (years) | 9.1 (−2.4; 20.5) | **−16.4 (−26.3; −6.5)** |
| Maternal educational levels | Middle level as reference, n = 244 (45.5%); |  |  |
|  | Low level, n = 84 (15.7%); | −10.1 (−23.9; 3.8) | −9.0 (−21.0; 2.9) |
|  | High level, n = 208 (38.8%) | −1.9 (−12.2; 8.5) | 6.2 (−2.8; 15.2) |

*Bold: p value<0.05. Linear regression analyses were used.*
